# Biomolecular solution X-ray scattering at the National Synchrotron Light Source

**DOI:** 10.1107/S0909049510036022

**Published:** 2010-11-05

**Authors:** Marc Allaire, Lin Yang

**Affiliations:** a National Synchrotron Light Source, Brookhaven National Laboratory, Upton, NY 11973, USA

**Keywords:** SAXS, WAXS, beamline, proteins, DNA/RNA

## Abstract

In recent years there has been a growing interest in the application of X-ray scattering techniques to biomolecules in solution. At NSLS, a new undulator-based beamline, X9, has been constructed to address the oversubscribed user demand for X-ray scattering. Beamline X9 has the capability to perform small/wide-angle X-ray scattering (SAXS/WAXS) all in one single instrument. This is accomplished by utilizing a vacuum sample/detector chamber that is an integral part of the SAXS scattering flight path. This vacuum chamber allows a WAXS detector to be positioned at a close distance from the sample, while not interfering with scattered X-rays at small angles from reaching the SAXS detector. A regular training program, the X9 workbench, has also been established to allow users to become familiar with beamline X9 for solution X-ray scattering.

## Abstract 2

A new beamline for simultaneous SAXS/WAXS of biomolecules in solution is described.

## 1. Introduction

Biomolecular solution X-ray scattering is becoming a mature technique for structural biology research. Recent developments in data analysis methods have presented an array of possibilities for extracting structural information from X-ray scattering data (see numerous recent reviews, *e.g.* Mertens & Svergun, 2010 ▶; Rambo & Tainer, 2010 ▶; Jackques & Trewhella, 2010 ▶). In addition to the characterization of basic geometric parameters such as the radius of gyration, comparison of experimental solution scattering data and that calculated from high-resolution structures provides the means to validate crystal/NMR structures, to assess the results of molecular dynamics simulations, and to construct rigid-body models of multi-subunit molecular complexes. Computation methods developed in the past decade now allow structural biologists to obtain *ab initio* low-resolution shape envelopes of molecules from solution scattering data. These low-resolution envelopes, used in coordination with X-ray crystallography, NMR and cryo-electron microscopy, have provided structural and functional insights into numerous systems. Methods for the use of solution scattering data and low-resolution shape envelopes as constraints on models constructed from NMR data and sequence-based structural prediction are being aggressively pursued. While solution scattering measurements have traditionally focused on small scattering angles that correspond to length scales relevant to the overall shape of the molecule, recent studies have begun to explore the information embedded in scattering data at wider angles pertinent to secondary and tertiary structures (Makowski, 2010 ▶). Such information may be useful for classifying protein structure into fold families, and as a sensitive tool to detect small conformation changes, for instance owing to ligand binding, that are not visible in small-angle scattering.

## 2. NSLS beamline X9

**Figure 1:**
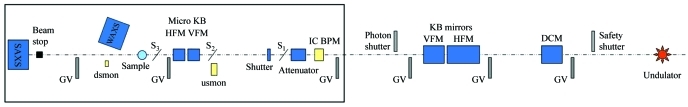
Schematic of the principal components of the NSLS beamline X9 from the undulator source (at the right) to the endstation hutch (large black box). Abbreviations used: gate valve (GV); double-crystal monochromator (DCM); Kirkpatrick–Baez (KB); horizontal/vertical focusing mirrors (HFM/VFM); ion chamber beam-position monitor (IC BPM); slits (S_1_, S_2_, S_3_); upstream/downstream monitor (usmon/dsmon).

Recent expansion of the use of solution X-ray scattering has resulted in a rapid increase in publications reporting the use of this technique. This has been driven by the availability of appropriate synchrotron beamlines and novel, informative and convenient software. In response to the growing demand of X-ray scattering, the National Synchrotron Light Source (NSLS) has commissioned a new undulator-based beamline, X9 (Fig. 1 ▶), with 10% of the beam time dedicated to the study of biomolecules in solution.

### 2.1. X-ray source and optics

**Figure 2:**
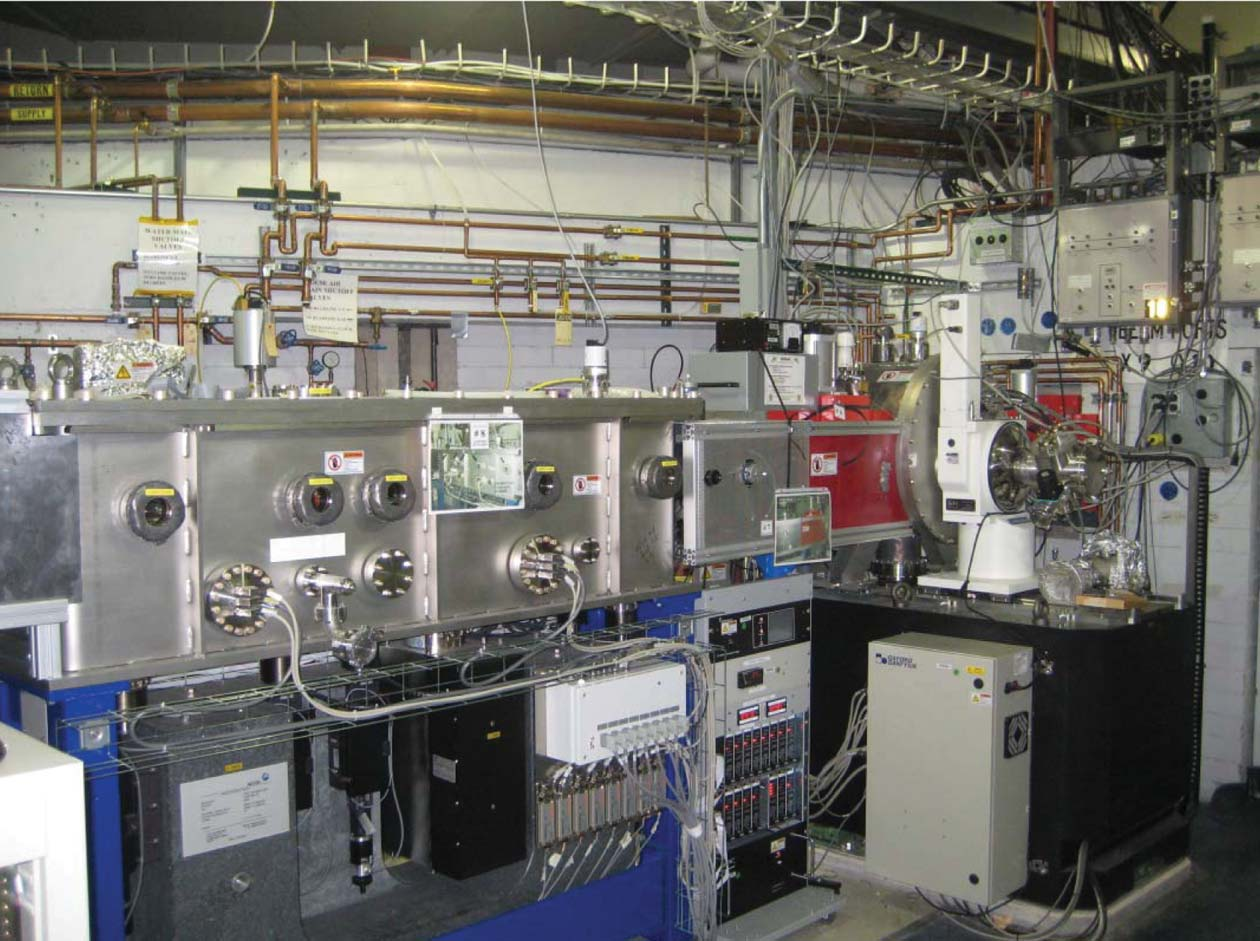
Photograph of the beamline optical components with the double-crystal monochromator (on the right) and the KB horizontal/vertical bimorph adaptive focusing mirrors (on the left).

The NSLS X-ray ring operates at 2.8 GeV and a maximum current of 300 mA. The photon source for beamline X9 is a mini-gap undulator (MGU) of length 360 mm with 23 × 14.5 mm periods and a minimum gap separation of 3.3 mm. The MGU characteristics were chosen to provide with the different harmonics a continuous spectrum with a fundamental energy of ∼2 keV to cover the phosphorus edge. The beamline optics (Fig. 2 ▶) consists of a cryocooled double-crystal monochromator located at 13.5 m from the source. A pair of 850 and 400 mm-long Kirkpatrick–Baez (KB) adaptive bimorph mirrors located at 15.5 and 16.3 m from the source are used to focus the horizontal and vertical monochromated X-ray beam, respectively. Two pairs of slits inside the hutch are used to define the beam size and divergence at the sample. A third pair of slits (guard slits) are mounted immediately upstream of the sample. Additional KB microfocusing mirrors could be used to deliver a beam size down to 10 µm.

### 2.2. Experimental station

**Figure 3:**
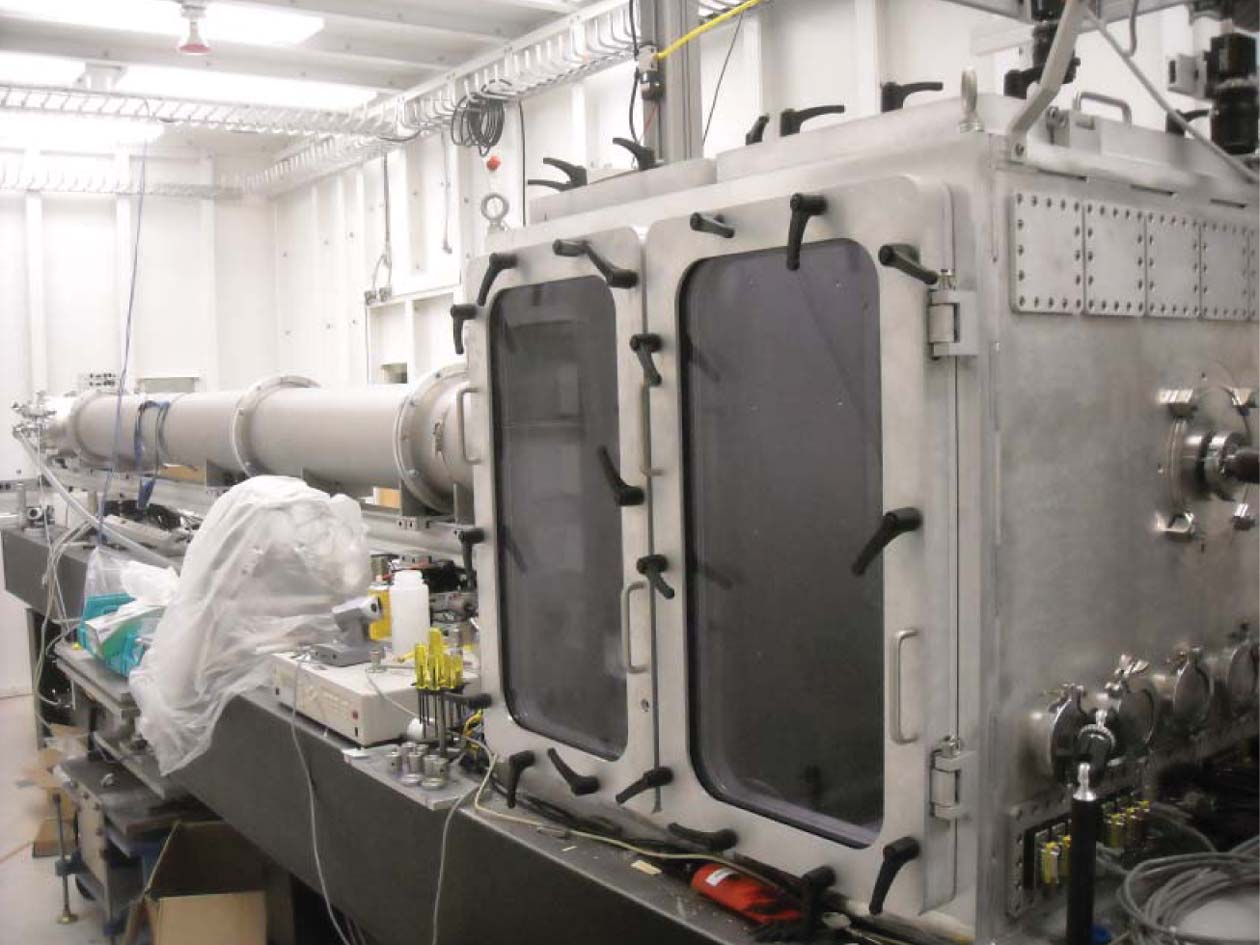
The long vacuum-compatible chamber of NSLS beamline X9 that contains both the SAXS (at the far end) and WAXS detectors (designed by Scott Coburn).

**Figure 4:**
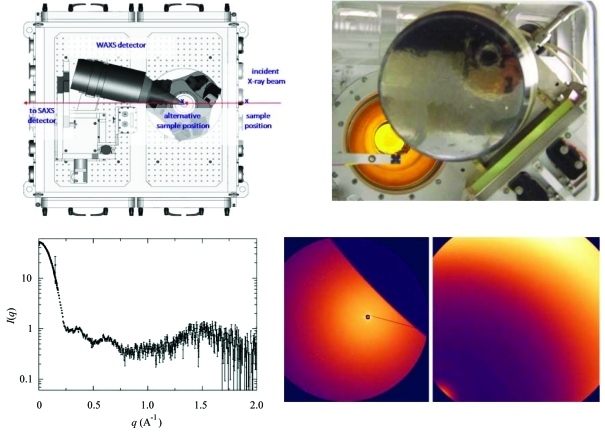
The NSLS X9 beamline was especially designed to acquire both SAXS and WAXS X-ray scattering data from a single exposure. The top-left panel indicates the position of the WAXS and the SAXS detectors relative to the X-ray beam. The top-right panel is a view from the X-ray beam in the direction of the detectors. This view indicates the overlapping region, which can be seen from the SAXS images (bottom-right panel, left image) cover by the WAXS detector (bottom-right panel, right image). The SAXS and WAXS data overlap between ∼0.12 and 0.2 Å^−1^ and are background-subtracted and combined automatically using beamline software, as shown by the data collected from a 3.7 mg ml^−1^ lysozyme solution (bottom-left).

**Figure 5:**
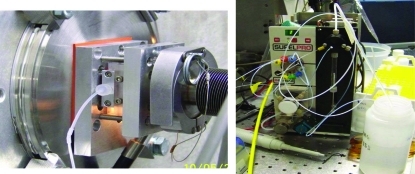
The solution sample cell at NSLS beamline X9 using a 1 mm glass capillary sealed across the vacuum path (left). The sample cell is connected through standard fittings to a Kloehn Versa 3 syringe pump (right).

The beamline endstation consists of a 5 m-long vacuum-compatible chamber (Fig. 3 ▶). The chamber was specially designed for the acquisition of small- and wide-angle X-ray scattering data with a single exposure (Fig. 4 ▶). X9 currently uses a custom-designed Photonic Science CCD detector as the WAXS detector and a Mar 165 CCD as the SAXS detector. For biological SAXS experiments the Mar detector is located 3.4 m from the sample. Together, these two detectors cover a combined *q* range of 0.005–2.0 Å^−1^, with overlapping data between ∼0.12 and 0.2 Å^−1^. The lowest *q* of 0.005 Å^−1^ at 12 keV is sufficient for biological solution X-ray scattering experiments. Currently the solution sample cell uses a 1 mm glass capillary sealed across the vacuum path, eliminating additional windows and air space that can produce background scattering (Fig. 5 ▶). The sample cell is connected through standard fittings to a Kloehn Versa 3 syringe pump.

## 3. Sample loading and data collection

Loading a sample is currently performed manually in a series of steps but we expect in the near future to have this process fully automated. Currently, after washing the sample cell by repeatedly pumping water through it, the tube connecting the sample cell to the pump is separated by ∼10 cm from the sample cell and the cell emptied by flushing nitrogen gas through the tube stem on the cell side. The users load a small air gap (5 µl) in the tube on the pump side to prevent any mixing of the sample solution with the water-containing tube. Then, the solution of interest, 20–100 µl, is loaded into the tube using the syringe pump. The tubes are then reconnected and the sample is pushed into the capillary by a known amount of tubing dead volume. Fine adjustment is made to assure that the meniscus of the sample has passed the position of the beam. The sample position is monitored with a video camera. For data collection, the user selects the exposure time, typically 60 s, and how much volume (∼20 µl) to flow though the capillary during exposure. The sample is flowed through the capillary during data collection in order to minimize radiation damage. The data collection command starts the pump at a given flow rate and then initiates data acquisition on both the SAXS and WAXS detector during X-ray exposure.

## 4. Data processing of SAXS/WAXS

Data processing is accomplished using a Python-based package developed at X9. This package provides the function to convert two-dimensional images into one-dimensional scattering profiles, perform buffer scattering subtraction and merge the SAXS and WAXS data. All these steps can be completed by running a single Python script at the command line. The data conversion is based on calibration of sample-to-detector distances using a silver behenate standard and is straightforward for the SAXS data. For WAXS data, because the detector is tilted at an angle of ∼20° to maximize the overlap between SAXS and WAXS data, this special geometry has to be taken into account when converting the detector pixel position into *q* values. Details of the WAXS detector and the data conversion will be reported elsewhere. The beam stop used in solution scattering experiments is semi-transparent to X-rays. Therefore the direct beam is always recorded in the SAXS scattering pattern. The Python code integrates the intensity within the direct beam spot and uses this value as the transmitted beam intensity to normalize scattering intensities of sample and buffer when subtracting buffer scattering. Since all measurements are performed using the same sample cell, the beam path length through the sample and empty cell scattering is expected to be constant. Therefore, by default, the script performs dark-current subtraction only. However, it can also be configured to subtract empty cell scattering. After separate background subtraction for the SAXS and WAXS data, the script automatically matches the SAXS and WAXS data within the overlapping region by multiplying the WAXS data by a constant factor. The script generates a plot of background-subtracted data so that the user can narrow down the *q* range within which the SAXS and WAXS data are merged, for example when the SAXS profile is very noisy at high *q*.

## 5. Beamline access

Beamline access is possible by submitting a biomolecular solution X-ray scattering proposal using the NSLS PASS system (http://www.nsls.bnl.gov/). The proposal is first reviewed by the beamline staff for feasibility, and then by external reviewers for its scientific merit. For X-ray scattering studies of biomolecular solution, rapid-access beam time proposals and requests have been implemented for quick turn­around. Successful proposals are allocated less than one day of beam time per request. We have also implemented a training program, the X9 SAXS workbench (http://www.nsls.bnl.gov/). The main purpose of this hands-on training program is to help build up the solution scattering user community at X9 and initiate participants to solution X-ray scattering. Through this workbench the participants learn the basic principles of solution scattering and how to collect solution scattering data at X9 on their own samples. We strongly recommend new users to attend this training program so they will already have solution scattering experience when they return to the beamline. During the workbench, participants are able to collect preliminary data on their own samples to be used to strengthen a regular beam time proposal for future measurements at X9.
